# Systemic inflammation biomarkers during angioedema attacks in hereditary angioedema

**DOI:** 10.3389/fimmu.2024.1400526

**Published:** 2024-06-17

**Authors:** Johana Gil-Serrano, Moisés Labrador-Horrillo, Paula Galvan-Blasco, Anna Sala-Cunill, Patricia Bigas, Javier Pereira-González, Olga Luengo, Victoria Cardona, Mar Guilarte

**Affiliations:** ^1^ Department of Allergy, Hospital Universitari Vall d’Hebron, Barcelona, Spain; ^2^ Allergy Research Unit, Institut de Recerca Vall d’Hebron (VHIR), Barcelona, Spain; ^3^ Department of Medicine, Universitat Autònoma de Barcelona, Barcelona, Spain

**Keywords:** hereditary angioedema, HAE-C1INH, HAE-FXII, inflammation, acute phase reactants, d-dimer, serum amyloid A, erythrocyte sedimentation rate

## Abstract

**Background:**

Hereditary angioedema (HAE) is a rare disease characterized by localized and self-limited angioedema (AE) attacks. A local increase of bradykinin (BK) mediates AE attacks in HAE, however the role of inflammation in HAE has been poorly explored We aim to analyze the role of inflammatory mediators in HAE patients during AE attacks.

**Methods:**

Patients with a confirmed HAE diagnosis due to C1 inhibitor deficiency (HAE-C1INH) or patients *F12* gene mutations (HAE-FXII) attending to our outpatient clinic between November-2019 and May-2022 were included. Demographic and clinical characteristics were analyzed. Blood samples were collected both during symptom-free periods (baseline) and during HAE attacks, and acute phase reactants (APR), such as serum amyloid A (SAA), erythrocyte sedimentation rate (ESR), C-reactive protein (CRP), D-Dimer and white blood cells were measured.

**Results:**

Seventy-eight patients were enrolled in the study, with a predominant representation of women (76%, n=59), and a mean age of 47.8 years (range 6–88). Among them, 67% (n=52) of patients had HAE-C1INH (46 classified as type 1 and 6 as type 2) while 33% (n=26) had HAE-FXII. During attack-free periods, the majority of patients exhibited normal levels of SAA, ESR, D-dimer, ACE and WCC. However, in a subset of patients (16% for SAA, 18% for ESR, and 14.5% for D-dimer), elevations were noted at baseline. Importantly, during HAE attacks, significant increases were observed in SAA in 88% of patients (p< 0.0001 vs. baseline), in ESR in 65% (p= 0.003 *vs.* baseline) and D-dimer in 71% (p=0.001 *vs*. baseline) of the patients. A comparison between baseline and acute attack levels in 17 patients revealed significant differences in SAA AA (p<0. 0001), ESR (p<0.0001) and D-dimer (p= 0.004). No significant differences were observed in CRP (p=0.7), ACE (p=0.67) and WCC (p=0.54). These findings remained consistent regardless of HAE type, disease activity or location of angioedema.

**Conclusion:**

The systemic increase in APR observed during HAE attacks suggests that inflammation extends beyond the localized edematous area. This finding underscores the potential involvement of inflammatory pathways in HAE and highlights the need for further investigation into their role in the pathophysiology of HAE.

## Introduction

Hereditary angioedema (HAE) is a rare genetic condition characterized by unpredictable episodes of subcutaneous or submucosal swelling attacks due to increased vascular permeability, secondary to the release of bradykinin (1–4). HAE can manifest with angioedema (AE) attacks in different locations, including the extremities, genitals, face, and gastrointestinal tract, as well as the upper airway, where they can be potentially life-threatening (1, 2, 4, 5).

HAE is divided into two types, HAE with C1 esterase inhibitor deficiency (C1INH) named HAE-C1INH, caused by mutations in the *SERPING1* gene, coding for C1INH and HAE without C1INH deficiency, known as HAE with normal C1INH (HAE-nC1INH) (1). HAE-C1INH is categorized into two subtypes: type 1, characterized by a quantitative deficiency of C1INH, and type 2, marked by a qualitative deficiency of C1INH. In HAE-nC1INH, several mutations have been described in genes coding proteins targeting the kallikrein-kinin system, such as factor XII (6, 7), plasminogen (8) or kininogen (9) and the vascular endothelium, such as angiopoietin (10), myoferlin (11) and heparan sulfate-glucosamine 3-O-sulfotransferase 6 (12). HAE-nC1INH due to mutations in the *F12* gene coding coagulation factor XII, is known as HAE-FXII and is the most frequent cause of HAE-nC1INH. Furthermore, in some patients no mutation has been described and are considered as HAE of unknown mechanism (HAE-UNK) (1).

HAE attacks initiate upon local activation of the contact system (CS) involving the interaction of the kallikrein kinin system (KKS), the fibrinolytic system and the coagulation system. FXII is activated (FXIIa) when it contacts with a negatively charged surface or macromolecules (1–5, 13). FXIIa cleaves the prekallikrein (PK) and the high molecular weight kininogen (HMWK) complex, which subsequently turns into plasma kallikrein (pK), and bradykinin (BK) is released (1–5). FXIIa and pK can additionally activate FXII in a positive feedback-loop, leading to an amplified response (2, 4, 5, 9, 13, 14).

BK is a vasoactive nonapeptide that acts on the surface of endothelial cells by binding to its B2 (mainly) and B1 receptors increasing vascular permeability, and subsequently resulting in AE attacks (2–5, 13, 14). BK is rapidly metabolized by several enzymes, such as angiotensin-converting enzyme (kininase II, ACE), carboxypeptidase N (kininase I), aminopeptidase P (APP), neutral endopeptidase (NEP), and dipeptidyl peptidase IV (DPPIV) (13–15).

Acute phase reactants (APR) are serum proteins that exhibit significant increases in various inflammatory conditions, whether acute or chronic (16–18). Commonly utilized APR include erythrocyte sedimentation rate (ESR), C-reactive protein (CRP), and serum amyloid A (SAA), which play crucial roles in the diagnosis and monitoring of numerous diseases such as severe bacterial or viral infections, as well as chronic inflammatory disorders like Crohn’s disease and rheumatoid arthritis (16–20). Additionally, in certain conditions, elevated levels of other APR such as haptoglobin, alpha-1-antitrypsin, C3, C4, hepcidin, or fibrinogen may also be observed (16–18). D-dimer is generated through the breakdown of the fibrin mesh by plasmin, an enzyme responsible for fibrinolysis. Additionally, D-dimer acts as an APR, triggering elevated levels of cytokines such as IL-6. Consequently, this reciprocal interaction suggests that D-dimer and other fibrin degradation products might impact inflammatory and acute-phase responses by fostering neutrophil and monocyte activation, thus stimulating the release of IL-6 (21–23).

When an HAE attack occurs, a local increase of BK has been described (13), however the role of inflammation in HAE was been poorly explored. Therefore, the aim of this study was to investigate the involvement of inflammatory mediators in patients with HAE during the acute attacks.

## Methods

### Study population

Patients with a diagnosis of HAE-C1INH or HAE-FXII attending the Angioedema Unit at the Allergy Department of Vall d’Hebron University Hospital (Barcelona, Spain) between November 2019 to May 2022 were prospectively recruited. Only patients who agreed to participate in the study and signed the informed consent were included. All patients with a HAE-FXII diagnosis had a genetic study confirming mutation in the F12 gene. HAE-C1INH was confirmed by terms of immunochemical and genetic study. Those who did not sign the informed consent, or who had been diagnosed with HAE-nC1INH with other mutations were excluded. The study was approved by the hospital’s ethics committee (project code PR (AG) 344/2019).

### Demographic and clinical data collection

Demographic and baseline clinical data regarding HAE, comorbidities, treatment, age of onset, locations and triggers of attacks were collected from electronic medical records. Data regarding acute AE attack such as location, severity, triggers, duration, and response to treatment was collected during the acute attack visit in a specific questionnaire. Finally, time since the last attack was also recorded.

### Sample collection and assessment of acute phase reactants

Baseline samples were obtained during symptom-free periods from peripheral blood prospectively from patients in the outpatient clinic during routine follow-up. To assess that the samples were collected during attack remission, samples were not obtained if the last attack had occurred during the previous 7–10 days. Acute blood samples were collected during angioedema episodes both in the outpatient clinic and in the emergency department (ED). Participants were instructed to come to the Hospital during an angioedema attack. Serum and citrated plasma samples were obtained and processed from patients with HAE-C1INH and HAE-FXII in symptom-free periods and during attacks and stored at −80°C until analyzed.

APR and inflammatory markers were measured according to the hospital’s routine techniques, normal ranges (lower-higher): SAA by nephelometry (normal range 0-7mg/dL), serum CRP by a particle-enhanced immunoturbidimetric assay (normal range 0.03-0.5mg/L), ESR levels by capillary photometry (normal range 0-20 mm/h), plasma D-dimer by immunoturbidimetric assay (normal range 0-243 ng/ml), ACE by spectrophotometry (normal range 13-63 U/L) and white blood cells count (WCC) by automated cell counting (normal range 4-11 x 10^9/L).

### Statistical analysis

**Figure 1:**
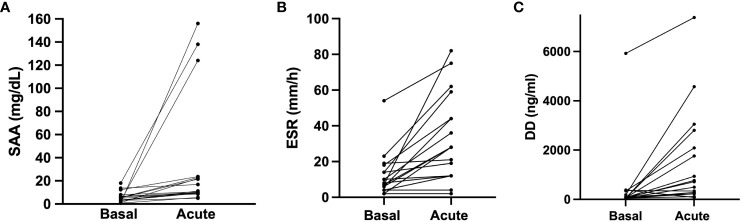
Acute phase reactants in baseline and during angioedema attacks. SAA, ERS and D-dimer acute levels were compared to their baseline levels in 17 HAE patients (12 HAE-C1INH and 5 HAE-FXII) who presented acute attacks. **(A)** Acute serum amyloid protein concentration median 11.3 (IQR 9.5–23.3) mg/dL *vs* basal median 4.9 (IQR 2.82–7.3) mg/dL in the same individuals (*p*<0.0001). **(B)** Dimer D concentration in acute median 718 (IQR 232–2447) ng/mL *vs* baseline median 71 (IQR 50–268) ng/mL in the same individuals (*p*=0.0004). **(C)** comparison of erythrocyte sedimentation rate (ERS) acute median 28 (IQR 12–51.5) mm/h *vs* basal median 8 (IQR 5–16) mm/h in the same individuals (p<0.0001). Data is expressed as median and interquartile range (IQR). Groups are compared using a Wilcoxon test. DD, dimer D; ESR, Erythrocyte sedimentation rate. SAA, serum amyloid A protein.

**Figure 2:**
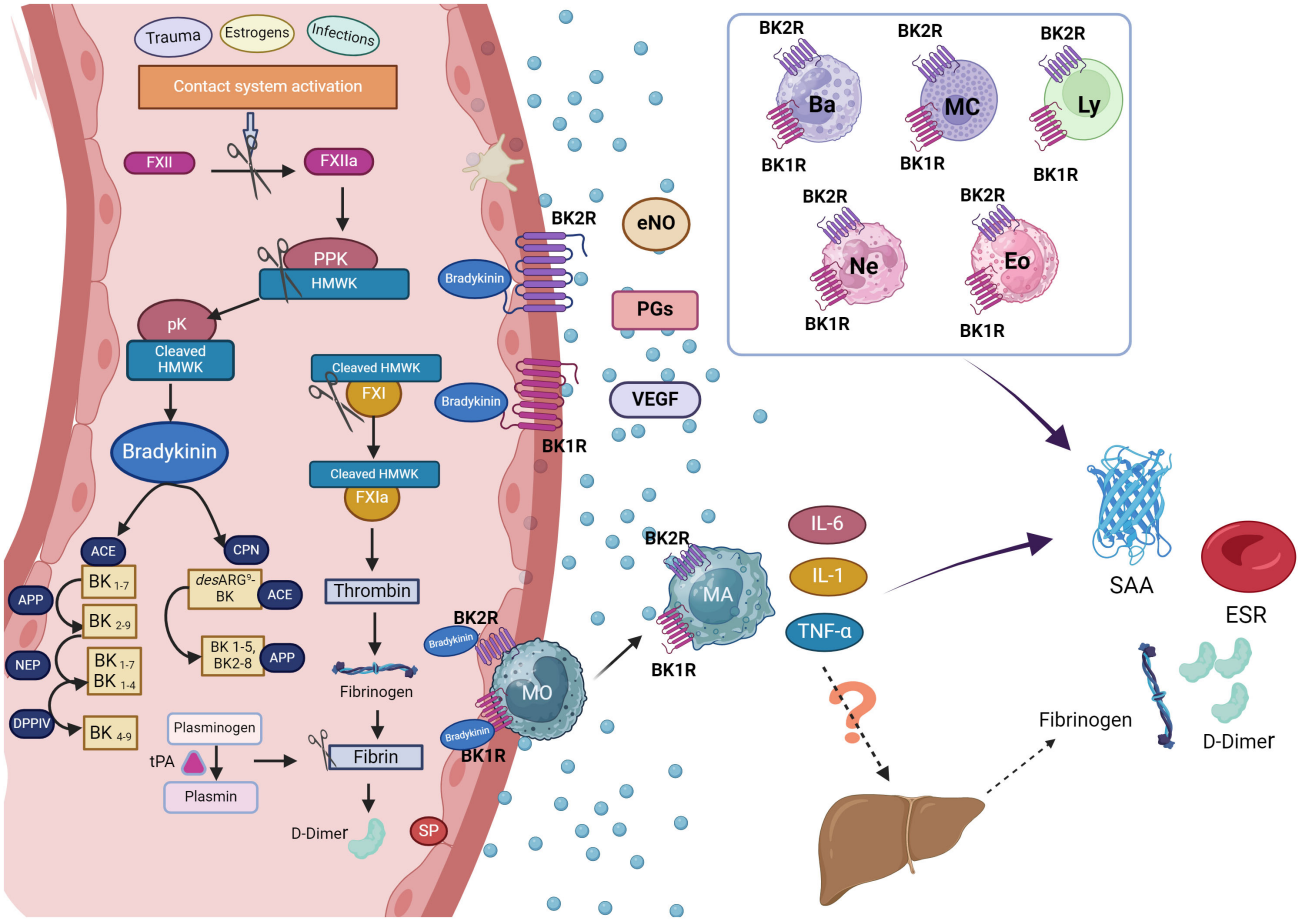
Hypothetical mechanism of acute phase reactants production in hereditary angioedema attacks. During hereditary angioedema (HAE) attacks, the release of bradykinin (BK) following activation of the contact system is a pivotal event, leading to angioedema when it binds to its receptors (BK1R and BK2R) on endothelial cell surfaces. During this process, D-dimer, a product of fibrinolysis, may also be generated. The liver is the primary source of acute phase reactants (APR), although as endothelial cells, macrophages, or other leukocytes can also contribute to their production. In HAE attacks, BK released upon contact system activation can also be bind to BK1R1 and BK2R expressed not only endothelial cells but also in eosinophils, basophils, neutrophils, mast cells and lymphocytes: This binding promotes the release of pro-inflammatory cytokines from these cells potentially contributing to inflammation. Consequently, this cascade of events could lead to an increase in acute phase reactants observed during HAE attacks. Figure created with BioRender.com. ACE, angiotensin-converting enzyme; APP, aminopeptidase p; Ba, basophil; BK1R, bradykinin receptor 1; BK2R, bradykinin receptor 2; CPN, Carboxypeptidase N; DPPIV, dipeptidyl peptidase IV; Eos, eosinophil; eNOs, Endothelial nitric oxide; ESR, erythrocyte sedimentation rate; FXII, factor XII; FXIIa, activated factor XII; FXI, Factor XI; FXIa, activated factor XI; HMWK, high molecular weight kininogen; IL-6, interleukin 6; IL-1, interleukin 1; Ly, lymphocytes; MA, macrophages; Mc, mastocyte; MO, monocytes; NEP, neutral endopeptidase; Ne, neutrophil; PGs, prostaglandins; PPK, Prekallikrein; pK, plasma Kallikrein; SAA, serum amyloid A protein; SP, Substance P; tPA, tissue plasminogen activator; TNF-α, Tumour Necrosis Factor α; VEGF, Vascular Endothelial Growth Factor.

Data collection was carried out according to previously established protocols and data were entered into an EXCEL database. Statistical analysis was analyzed with SPSS version 17 (SPSS Inc., Chicago, Ill., USA). Descriptive analysis of frequencies of the variables are expressed as median and interquartile range (IQR). The Student’s T test for paired data was used to compare the different serum/plasma biomarkers between the patient groups and between measurements at baseline and during attacks. Comparative analyses for quantitative variable data without a normal distribution were conducted using the Wilcoxon rank-sum test for comparisons of 2 groups. A two-sided p value of 0.05 or less was considered statistically significant. **Figure 1** was created with GraphPad Prism 9 (GraphPad Software, San Diego, California, USA) and **Figure 2** with BioRender.com (Toronto, Canada).

## Results

### Patient characteristics

A total of 78 patients were included. Patient’s characteristics are described in **Table 1**. Seventy-six percent were women (*n*=59) and 24% were men (*n*=19), with mean age 47.8 years (range 6–88). Sixty-seven percent (*n*=52) of patients had a diagnosis of HAE-C1INH (type 1 *n*=46 and type 2 *n*=6) and were classified as group 1; 33% (*n*=26) had a HAE-FXII and were classified as group 2. The median age of onset was higher for group 2 than for group 1, being 20 years (IQR 18–26 years, SD 12) vs 14 years (IQR 5.5–20 years SD 9.03) respectively. Attack location and triggers according to group are shown in **Table 1**.

**Table 1:** Patient characteristics.

	Group 1 (*n*=52)		Group 2 *(n*=26)	Total (*n*=78)
	HAE-C1INH type 1 (*n*=46)	HAE-C1INH type 2 (*n*=6)	HAE-FXII (n=26)	
**Age (median; range)**	47 years 6-88	35.5 years 21-51	52.3 years 21-79	Mean 47.8 years 6-88
**Female (n; %)**	*n*=30 65%	*n*=4 67%	*n*=25 96%	*n*=59 76%
**Age of disease onset (mean; range)**	14,57 years 1-40 years	10 years 6 months-22 years	24 years 16-75 years	17.07 years 6 months – 75 years
**Location of attacks**	Facial 43% (*n*=20) Peripheral 98% (*n=*45) Abdominal 98% (*n=*45) Genital 43% (*n*=20) Oropharynx 15% (*n*=7) Larynx 7% (*n*=3)	Facial 50% (*n*=3) Peripheral 100% (*n=*6) Abdominal 100% (*n=*6) Genital 50% (*n*=3) Oropharynx 33% (*n*=2)	Facial 85% (*n*=22) Peripheral 54% (*n=*14) Abdominal 62% (*n=*16) Oropharynx 15% (*n*=4)	Abdominal 86% (*n*=67) Peripheral 83% (*n*=65) Facial 58% (*n*=45) Genital 29% (*n*=23) Oropharynx 17% (*n*=13) Larynx 4% (*n*=3)
**Autoimmune comorbidities**	Antiphospholipid syndrome (*n*=1), autoimmune hypothyroidism (*n*=1) Wells syndrome (*n*=1)	Ankylosing spondylitis (*n*=1)	Autoimmune hepatitis (*n*=1)	Autoimmune/inflammatory diseases 6% (*n*=5)
**Triggers by frequency**	Trauma 60% (*n*=31) Stress 56% (*n*=29) Infections 27% (*n*=14) Oral contraceptives 6% (*n*=3) Ovulation 6% (*n*=3) Menstruation 13% (*n*=7) Pregnancy 6% (*n*=3) Others 15% (*n*=8)		Oral contraceptives 85% (*n*=22) Trauma 38% (*n*=10) Infections None Pregnancy 38% (*n*= 10) Stress 15% (*n*=4) Ovulation 8% (*n*=2) Menstruation 8% (*n*=2)	Trauma 52% (*n*=41) Stress 42% (*n*=33) Oral contraceptives 32% (*n*=25) Infections 18% (*n*=14) Pregnancy 17% (*n*=13) Ovulation 6% (*n*=5)

HAE-C1INH, hereditary angioedema due to C1 inhibitor deficiency; HAE-FXII, hereditary angioedema with mutations in factor XII.

In order to study whether APRs were increased due to other conditions rather than HAE, autoimmune and/or inflammatory diseases were assessed. In group 1, four patients (8%) (**Table 1**) had a diagnosis of antiphospholipid syndrome, autoimmune hypothyroidism, Wells syndrome or ankylosing spondylitis respectively. A very slight elevation of baseline APR was observed only in two patients. One with ankylosing spondylitis had an ESR =34 mm/h (normal range <20mm/h) and CPR=0.9 mg/dL (normal range <0.5 mg/dl), and the patient with an antiphospholipid syndrome had an ESR=21 mm/h, CPR=0.7 mg/dL and SAA=8 mg/dL (normal range <7 mg/dL). In group 2, only one patient (4%) was diagnosed with autoimmune hepatitis, with an ESR >20mm/h (37mm/h) and CPR >0.5mg/dL (0.7mg/dL). None of the patients in either group1 or 2 reported any thromboembolic events.

At the time of inclusion, 31 patients with HAE-C1INH (group 1) were only on-demand therapy (ODT) and 21 under long-term-prophylaxis (LTP). Of these patients, 11 were treated with plasma-derived C1INH concentrate (pd-C1INH), 5 patients with lanadelumab and 5 patients received treatment with attenuated androgens. In group 2 (HAE-FXII), 4 patients were under LTP with tranexamic acid (3 patients) and with pdC1INH (one patient).

### Acute phase reactants

Baseline samples were obtained in 78 patients and acute samples were collected during acute attacks in 17 patients (12 with HAE-C1INH and 5 with HAE-FXI) (**Table 2**). Seven blood samples were obtained during facial attacks (none of them had submucosal involvement), 6 during abdominal attacks, and 4 during peripheral attacks. One patient presented a concomitant peripheral and abdominal attack. Twenty-nine percent of the attacks were mild, 47% moderate and 24% were severe. The median time from the onset of the attack until the sample collection was of 12 hours (IQR 5–18, SD 7.2). Fifteen patients had treated their attack with icatibant 30 mg sc before the sample collection.

**Table 2:** Acute phase reactants in baseline and during acute attacks.

Biomarker	SAA (mg/dL)	ESR (mm/h)	CPR (mg/dL)	D-dimer ng/mL	WCC (x10E9/L)	ACE (U/L)
**Basal; median (IQR)**	3.57 (2.6-6.75)	14 (7-23)	0.2 (0.04-0.32)	81 (52-179.75)	5.8 (5,44-11,19)	27.5 (22-42.6)
**Acute attack;** **median (IQR)**	11.3 (9,5-23.3)	28 (12-51.5)	0.56 (0.16-1.4)	718 (253-2447)	5.8 (5.66-8.39)	27.7 (21.2-48.7)
***p* value**	*p* = 0.009	*p* =0.003	*p =* 0.779	*p* =0.011	*p =* 0.594	*p*=0.43

Basal SAA was obtained in 55 and acute SAA in 17 patients; basal ESR in 65 and acute ESR in 17 patients, basal CRP in 56 and acute CRP in 17 patients; basal D-dimer in 62 and acute D-dimer in 17 patients; basal WCC in 77 and acute WCC in 17 patients and basal ACE in 59 and acute ACE in 17 patients. p value between basal levels vs acute levels (T student).ACE: Angiotensin-converting enzyme (U/L), normal range: 13-63 U/L; CRP: C-reactive protein (mg/dL), normal range: 0.03-0.5 mg/dL; D-Dimer (ng/mL), normal range: 0-243 ng/mL; ESR: Erythrocyte sedimentation rate (mm/h), normal range: 0-20 mm/h; SAA: Serum amyloid A. Normal range: 0-7mg/dL; WCC: White cell count (x10E9/L) normal range:4.00-1x10E9/L.

At baseline (group 1 and 2) most patients had normal values of the evaluated APR. However, in 16% (9/55), 18% (14/65) and 14.5% (9/62) of patients, an elevation of SAA, ESR, and D-dimer respectively was observed. These values were not related with HAE type. Basal CRP was elevated in only one patient, whereas ECA and WCC were not increased at baseline conditions. Median levels of APR are shown in **Table 2**.

During acute attacks, SAA was elevated in 88% of the patients (12 HAE-C1INH and 3 HAE-FXII) with a median of 16.8 mg/dL (IQR 9.9–23.7); ESR in 65% (7 HAE-C1INH and 4 HAE-FXII), median 44 mm/h (IQR 28–62); and D-dimer was increased in 71% (9 HAE-C1INH and 3 HAE-FXII), median 938 mg/mL (IQR 417-2930). By contrast, CRP was slightly elevated in only 3 HAE-C1INH patients (18%), median 1.4 mg/dL (IQR 1–15.2), and no changes were observed in ACE levels or in WCC. Increases of APR were not dependent on angioedema location, although abdominal attacks tended to increase SAA (p =0.08) while ESR tended to be higher in facial attacks (p=0.09). ASR levels were higher in moderate and severe attacks, although non-significant (p>0.05). Trigger of attacks was only recognized in five patients, traumatism in 3 and stress in 2. APR in these patients were not higher than in angioedema attacks with an unknown trigger. Individual patient and attack characteristics, and baseline and acute APR levels of are shown in **Table 3**. APR levels did not differ in the two patients with a concomitant disease.

**Table 3:** Characteristics and acute phase reactants levels in patients with acute attacks.

Patient	Age	Sex	HAE type	LTP	Concomitant Disease	Attack			ESR		SAA		Dd		CPR		WCC	
						Location	Treatment	Severity	Basal	Acute	Basal	Acute	Basal	Acute	Basal	Acute	Basal	Acute
**1**	29	F	HAE-C1INH	No	None	Abdominal peripheral	Yes	Severe	7	**28**	2,6	**124**	188	**4576**	0,2	**15,84**	5,9	5,7
**2**	54	F	HAE-C1INH	No	None	Abdominal	Yes	Severe	**23**	**62**	3,05	**11,3**	71	**1762**	0,4	0,33	9,1	6,7
**3**	25	F	HAE-C1INH	pdC1INH	None	Abdominal	Yes	Moderate	2	2	3,11	**9,27**	50	**764**	0,3	0,5	5,4	5,8
**4**	48	F	HAE-C1INH	No	Autoimmune Hypothyroidism	Abdominal	Yes	Severe	14	**59**	4,91	**9,94**	73	**718**	0,03	0,03	11,2	5,6
**5**	49	F	HAE-C1INH	pdC1INH	None	Abdominal	Yes	Severe	6	**44**	12,3	**23,7**	**362**	**342**	0,08	0,15	5,3	5
**6**	48	F	HAE-C1INH	No	None	Facial	No	Mild	**54**	**75**	6,6	**9,94**	53	116	0,31	**1,27**	11,2	10,3
**7**	24	F	HAE-C1INH	No	None	Facial	Yes	Mild	4	12	**8,04**	**10,5**	**5928**	**7382**	0,5	0,02	13	9,6
**8**	61	F	HAE-C1INH	No	None	Peripheral	Yes	Moderate	10	**36**	5,2	**21,5**	50	**3054**	0,02	0,03	5,3	5,8
**9**	56	M	HAE-C1INH	No	None	Peripheral	Yes	Moderate	4	4	1,21	**22**	50	**2803**	0,1	0,03	4,8	4,9
**10**	26	M	HAE-C1INH	No	None	Peripheral	Yes	Moderate	18	12	18	**138**	73	**938**	0,12	**1,54**	9,5	10,4
**11**	53	F	HAE-C1INH	Danazol	None	Peripheral	Yes	Moderate	6	**28**	6,33	**8,55**	114	230	0,33	0,04	4,9	6,6
**12**	38	F	HAE-C1INH	No	None	Abdominal	Yes	Moderate	10	12	0,72	**156**	50	235	0,12	0,1	10,2	9,9
**13**	26	F	HAE-FXII	No	None	Facial	Yes	Mild	2	**28**	0,72	5,6	50	**283**	0,2	0,12	9,6	9,6
**14**	43	F	HAE-FXII	No	None	Facial	Yes	Mild	8	**44**	**13,7**	**16,8**	58	**493**	0,14	0,16	5,6	7,2
**15**	79	F	HAE-FXII	No	Multiple myeloma	Facial	Yes	Mild	19	**21**	3,5	**9,67**	50	223	0,16	0,2	4,8	5,8
**16**	44	F	HAE-FXII	No	None	Facial	Yes	Moderate	14	19	5,59	**22,9**	**379**	83	0,03	0,02	3,9	4,8
**17**	51	F	HAE-FXII	No	None	Facial	No	Moderate	7	**82**	3,08	5	**348**	**2091**	0,2	0,19	6,2	5,8

In bold are shown acute phase reactants with levels above normal range. ESR: normal range 0-20 mm/h, SAA: normal range 0-7mg/dL, CPR normal range 0.03-0.5mg/L, DD normal range 0-243 ng/ml, WWC normal range 4-11 x 10^9/L.. HAE-C1INH, hereditary angioedema due to C1 inhibitor deficiency type 1 or 2; HAE-FXII, hereditary angioedema due to mutations in FXII gene; LTP, long term prophylaxis; DD, D dimer; ESR, Erythrocyte sedimentation rate; SAA, serum amyloid A protein; WCC, white blood cells count.

Interestingly, upon analyzing the results from patients for whom both baseline and acute samples were available, we observed a significant increase in SAA (4.9 (2.8–7.3) *vs*. 11.3 (9.5–23.3) mg/dL; basal *vs*. acute; median (IQR); p< 0.0001) (**Figure 1A**), in ESR (8 (IQR 5–16) *vs.* 28 (12–51.5) mm/h; basal *vs*. acute; median (IQR); p<0.0001) (**Figure 1B**) and in D-dimer levels (71 (50–268) *vs.* 718 (232–2447) ng/mL; basal *vs*. acute; median (IQR); p=0.004) (**Figure 1C**). These results reinforce the concept that some APR may indeed increase during HAE attacks. Notably, no significant differences were observed in CRP (p=0.7), ACE (p=0.67) and WCC (p=0.54). And again, these results were consistent irrespective of HAE-C1INH or HAE-FXII subtype.

None of the patients recalled fever nor other symptoms that could suggest viral or bacterial infection or any inflammatory process either in baseline or during angioedema attacks. This is consistent with the fact that WCC remained within normal values in both conditions.

## Discussion

This study shows, that conventional inflammatory pathways may be involved in the pathophysiology of acute AE attacks in HAE. Despite the limited acute sample size, we have demonstrated a substantial increase of some APRs such as SAA, ESR and D-dimer during AE attacks, both in HAE-C1INH and in HAE-FXII patients.

Coagulation parameters and markers of activation of the fibrinolytic pathway, such as D-dimer and the contact system had been previously explored in HAE-C1INH (21, 24, 25). In agreement with findings from previous studies (21, 24, 25), we have observed an increase of D-dimer during attacks in HAE-C1INH patients. Noteworthy, this study shows for the first time that AE attacks can also increase D-dimer in HAE-FXII. D-dimer is produced during the breakdown of the fibrin mesh by plasmin. Elevation of D-dimer in HAE-C1INH attacks may mean secondary hyperfibrinolysis, rather than hypercoagulation, because plasminogen levels during baseline periods did not differ from those recorded during AE attacks and are similar to the healthy control levels (24). In a study of 79 patients with HAE-C1INH, 80% of them presented an increase in D-dimer during AE attacks, that was higher in patients with angioedema affecting the submucosa (abdominal and oropharyngeal-laryngeal) compared to subcutaneous (peripheral and facial) (25). Nevertheless, severity and activity of HAE-C1INH was not considered. In our study, apart from abdominal attacks, we have also observed an increase of D-dimer during facial and peripheral attacks. However, these differences should be evaluated cautiously and could be related to the small sample size. In our study, D-dimer was elevated in symptom-free periods only in 14.5% of patients. One study showed a significant elevation of D-dimer at baseline compared with controls (24). This discrepancy can be due to the different study design, since we have considered an elevation of D-dimer when values were over the normal threshold, but we have not compared patients with controls. In addition to fibrinolysis contributing to the generation of D-dimer in HAE patients via contact system activation, it is crucial to underscore that inflammatory states trigger the production of fibrinogen in response to interleukin-6 (IL-6), interleukin-1 (IL-1), and tumor necrosis factor (TNF) (18). This reciprocal interaction suggests that D-dimer and other fibrin degradation products might impact inflammatory and acute-phase responses by promoting neutrophil and monocyte activation, thus facilitating the release of IL-6 (22, 23). In HAE attacks, an elevation of IL-6 has been demonstrated (26), so it is plausible to hypothesize that the activation of the inflammatory cascade and the production of fibrinogen by endothelial and inflammatory cells in HAE patients can contribute to the production of D-dimer (18). Nevertheless, additional research is required to validate this hypothesis.

APRs are known to respond to pro-inflammatory cytokines, such as IL-1, IL-6 or tumor necrosis factor-alpha (TNF-α), and to the local production of endothelial nitric oxide (NO), prostaglandins, and endothelial adhesion molecules (16–18). Although the liver is the main producer of APR, other non-hepatic sources such as endothelial cells and macrophages have been identified (18–20). SAA, which belongs to the apolipoprotein family, is produced mainly in the liver and its secretion reasonably increases during inflammatory states. *In vitro* IL-6 has a synergism in the production of SAA either in combination with IL-1β or with TNF-z (27). SAA is present constitutively in plasma, and the increase in SAA levels observed in our patients during AE attacks could be in response to the local production of proinflammatory cytokines (IL-6, IL1-β and TNF-z) independently of the hepatic production, as proposed in **Figure 2**.

ESR, is an indirect measure of the amount of fibrinogen produced during inflammatory states, such as infections, in malignancy or other immune diseases (16–18).

We have noted a rise in ESR among a subset of our patients during symptom-free intervals. Notably, during acute attacks, a substantial proportion of our patients experienced a significant increase in ESR, indicating the occurrence of systemic inflammation. Until now, no prior study had investigated the role of ESR during HAE acute episodes. While we did not measure fibrinogen levels, a plausible explanation for our observations could be that, following the activation of the contact system and fibrinolysis, there is a localized production of fibrinogen during attacks. In angioedema due to ACE inhibitors (ACEi-AE), a type of AE that, like HAE-C1INH and HAE-FXII, is mediated by BK, a 1.5-fold increase in fibrinogen has been observed during attacks (28). Moreover, fibrinogen is able to increase the vasodilator potency of BK by 10-fold and to increase bradykinin-induced vasodilator-stimulated phosphoprotein phosphorylation (28). This potentiation of bradykinin-induced vasodilation suggest that fibrinogen might contribute to the pathophysiology of ACEi-AE. An elevation of CRP has also been described in ACEi-AE (29–31). CRP, is another APR, mainly produced in the liver, widely used as a biomarker in inflammatory diseases, that increases mostly in response to microbial components during infections and in autoimmune diseases (18, 32–34). CRP primarily responds to IL-6 and, to a lesser extent, to IL-1β and TNF-α (18, 33). In accordance with a previous study in 25 HAE-C1INH patients (35), we found that CRP levels were within normal range during baseline and remained unaltered during HAE attacks. On the other hand, during HAE attacks, levels of IL-1, IL-6 and TGF-b have been found to be significantly higher compared to remission and those from healthy controls, whereas no activation of liver CPR production has been observed (26). Furthermore, it has been established that CRP may be produced extrahepatically by various tissues, including neurons, adipose tissue, lungs, kidneys, and intestines (33, 34). In fact, in our study, CPR remained unaltered even in abdominal attacks, so further studies are needed to elucidate the reason for the lack of CRP production during AE attacks in HAE patients compared to ACEi-AE (29).

We postulate that in individuals with HAE, the heightened levels of SAA, ESR and D-dimer during acute attacks could be a result of the localized production of proinflammatory cytokines. Specifically, SAA and ESR may be increasing locally in response to the release of IL-6, IL-1β, and TNF-α, rather than systemic proinflammatory cytokines, suggesting a limited hepatic APR production. This discrepancy might explain the absence of CRP, which is primarily produced in the liver. Moreover, various immune cells, including eosinophils, basophils, neutrophils, mast cells, and lymphocytes, express BK1 and BK2 receptors on their surfaces (36). This cellular composition suggests a possible pivotal role in the inflammatory process during HAE attacks, as these cells could be activated through BK (5). Consequently, in patients with HAE, bradykinin may be implicated in the local production of APR. **Figure 2** illustrates how the release of bradykinin during HAE attacks may contribute to acute phase reactant production through immune cell activation and subsequent cytokine release, highlighting the complex interplay between inflammation and the contact system in HAE pathophysiology.

Reliable biomarkers for distinguishing between various types of HAE are currently scarce, with genetic studies being a primary means of differentiation. Assessment of C4 levels and C1INH activity and function remains crucial in delineating HAE types (1, 2). Furthermore, there is a notable gap in potential biomarkers for discerning between different types of angioedema (e.g., mast cell-mediated or bradykinin-induced) (1, 2). Bradykinin has been proposed as a potential biomarker, but its rapid metabolism and the technical challenges associated with its measurement make it impractical for routine use (37–39). Other groups have put forth alternative candidates. The potential use of HMWK and the plasma PK-HK complex has been explored (37), although standardization of techniques for their quantitation remains a hurdle, and they have not yet been incorporated into routine clinical practice. Elevated levels of adrenomedullin and urokinase plasminogen activator (uPAR) during acute attacks, measured using transcriptomics, have also been suggested as potential biomarkers, although these techniques require further standardization before widespread use (40). Finally, Tie-2 (Angiopoietin-1 receptor), fibroblast activation protein-alpha (FAP-α), and tissue plasminogen activator (tPA) have also been postulated as biomarkers to distinguish between different types of AE (41). However, it is crucial to acknowledge that none of these biomarkers have yet been incorporated into routine clinical practice, and their measurement requires complex techniques. SAA, ESR, and D-dimer, being easily measurable, could be regarded as for AE biomarkers. Nevertheless, a comprehensive analysis is required to understand the role of APR in other types of angioedema. High SAA levels were found in 67 patients with chronic spontaneous urticaria (CSU), with a particularly significant elevation in those with both CSU and AE (42). Furthermore, in anaphylaxis, mast cell activation has been linked to contact system activation (43, 44), and in CSU, an increase in plasma D-dimer has been reported, correlating with disease activity (45–47). Although still an unexplored area, it is conceivable that in mast cell-mediated angioedema, APR could also see an increase during acute episodes.

To our knowledge, this study shows for the first time that SAA and ESR are elevated in HAE-C1INH and in HAE-FXII attacks and D-dimer is elevated also in HAE-FXII attacks, since the elevation in AE attacks in patients with C1INH deficiency has been previously described. Clearly, large scale follow-up studies are needed to elucidate the behavior and clinical usefulness of SAA and ESR in different HAE types.

### Limitations of the study

Several limitations must be considered in interpreting our findings. These include the small sample size, absence of a control group for comparative analysis (including other inflammatory diseases), absence of monitoring changes of APR over time and the lack of assessment for other relevant APRs such as IL-6, fibrinogen, and alpha-1-antitrypsin. Moreover, there is a wide range in the time from the onset of the attack until the sample collection. Addressing these limitations through larger, well-controlled studies could enhance our understanding of HAE pathophysiology and improve diagnostic and treatment strategies for affected individuals.

## Conclusions

In conclusion, our study provides valuable insights into the inflammatory mechanisms involved during AE attacks in HAE patients. While these attacks are localized, the significant elevation of APRs suggests a broader inflammatory response. The causal relationship between increased APR levels and HAE attacks warrants further investigation.

## Data availability statement

The original contributions presented in the study are included in the article/supplementary material. Further inquiries can be directed to the corresponding author.

## Ethics statement

The studies involving humans were approved by Comité d’Ètica. Hospital Universitari Vall d’Hebron. The studies were conducted in accordance with the local legislation and institutional requirements. Written informed consent for participation in this study was provided by the participants’ legal guardians/next of kin.

## Author contributions

JG-S: Writing – review & editing, Writing – original draft, Visualization, Software, Project administration, Methodology, Investigation, Formal analysis, Data curation. ML-H: Writing – review & editing, Writing – original draft, Validation, Supervision, Methodology, Investigation, Conceptualization. PG-B: Writing – original draft, Writing – review & editing, Methodology, Investigation, Formal analysis. AS-C: Writing – original draft, Writing – review & editing, Project administration, Methodology. PB: Writing – original draft, Writing – review & editing, Methodology, Investigation, Formal analysis. JP-G: Writing – original draft, Writing – review & editing, Project administration, Investigation, Formal analysis. OL: Writing – original draft, Writing – review & editing, Validation, Supervision, Methodology. VC: Writing – original draft, Writing – review & editing, Visualization, Validation, Supervision, Methodology. MG: Data curation, Writing – review & editing, Writing – original draft, Validation, Supervision, Resources, Methodology, Investigation, Funding acquisition, Formal analysis, Conceptualization.
